# SpeckTackle: JavaScript charts for spectroscopy

**DOI:** 10.1186/s13321-015-0065-7

**Published:** 2015-05-09

**Authors:** Stephan Beisken, Pablo Conesa, Kenneth Haug, Reza M Salek, Christoph Steinbeck

**Affiliations:** European Bioinformatics Institute (EMBL-EBI), Wellcome Trust Genome Campus, Hinxton, CB10 1SD UK

**Keywords:** JavaScript, Charting library, Spectroscopy

## Abstract

**Background:**

Spectra visualisation from methods such as mass spectroscopy, infrared spectroscopy or nuclear magnetic resonance is an essential part of every web-facing spectral resource. The development of an intuitive and versatile visualisation tool is a time- and resource-intensive task, however, most databases use their own embedded viewers and new databases continue to develop their own viewers.

**Results:**

We present SpeckTackle, a custom-tailored JavaScript charting library for spectroscopy in life sciences. SpeckTackle is cross-browser compatible and easy to integrate into existing resources, as we demonstrate for the MetaboLights database. Its default chart types cover common visualisation tasks following the *de facto* ‘look and feel’ standards for spectra visualisation.

**Conclusions:**

SpeckTackle is released under GNU LGPL to encourage uptake and reuse within the community. The latest version of the library including examples and documentation on how to use and extend the library with additional chart types is available online in its public repository.

**Electronic supplementary material:**

The online version of this article (doi:10.1186/s13321-015-0065-7) contains supplementary material, which is available to authorized users.

## Background

The visualisation of spectra from different analytical platforms is an essential aspect of every web-facing resource. Web technology has penetrated deeply into modern data repositories and spectral databases, facilitating global data access and usage. Consequently, these databases have become an integral part of many processes and pipelines in various fields of research and development.

Spectroscopic methods such as mass spectroscopy (MS), infrared spectroscopy (IR) or nuclear magnetic resonance (NMR) spectroscopy are commonly used to identity chemical components [1]. Online databases aggregate data from these experiments and serve as sources of information of small molecules and reference spectra for the life sciences community [2].

Amongst other information, the Human Metabolome Database (HMDB [3]) contains ∼9,400 spectra of small molecule metabolites found in the human body, the Madison-Qingdao Metabolomics Consortium Database (MMCD [4]) contains empirical NMR data for ∼20,300 metabolites, and MassBank [5], a high-quality mass spectral database, has over 40,800 mass spectra. Other databases with spectral data include the Metabolite and Tandem MS Database Metlin [6], the Golm Metabolome Database [7], the Lipidomics Gateway LipidMaps [8] and many more [2].

Although the visualisation requirements – the expected ‘look and feel’ – for the majority of spectra from different spectroscopic methods are well established, a dominant lack of a reusable customisable spectra viewer for the life sciences is noticeable. Before data is downloaded and processed in standalone expert applications, browser-based visualisation tools facilitate data selection and quick data lookup. Currently, the databases listed above use their own embedded viewers and new databases continue to develop their own visualisation tools because existing viewers are potentially too hard to migrate or lack a particular function because they were specifically developed for their database and type of data.

To mitigate this problem and encourage reuse of existing solutions, we have developed SpeckTackle, a easy to tweak, custom-tailored JavaScript charting library for spectroscopy in life sciences. The visualisation library SpeckTackle is freely available and targeted at life science communities that deal with spectroscopic data such as coming from mass spectroscopy, infrared spectroscopy, or NMR and require a flexible data viewer. The library contains several default chart types, supports common functionality, e.g. for spectra overlays or tooltips, and is designed to be portable.

## Implementation

SpeckTackle is written in the interpreted programming language JavaScript. JavaScript is a powerful scripting language to develop cross-browser compatible software libraries. In combination with HTML5 in modern browsers, JavaScript is the language of choice to ensure portability and wide applicability interactive web-facing tools [9,10].

SpeckTackle depends on the *D3* (Data Driven Documents [11]) and *JQuery* JavaScript libraries, which are used in many websites. D3 simplifies the manipulation of the DOM (Document Object Model) and provides visualisation components, which form the base of the SpeckTackle charting library. JQuery is primarily used for HTML traversal and Ajax handling. Both libraries are assumed to be defined globally within the website as it is typically the case.

The charting library has been tested on recent versions of all major modern web browsers (Firefox, Internet Explorer, Opera, Chrome, and Safari) that adhere to HTML5 and SVG web standards, which in case of SVG have a global usage of about 90% (http://caniuse.com/#feat=svg). Compatibility to older browsers such as Internet Explorer 9 has been sacrificed in favour of source code comprehensibility and maintainability.

The project – including extensive documentation and example charts – is available online on the project repository BitBucket and a Mini-website is provided that is browsable from within the full text HTML version of the article [Additional file 1]. The source code is released under GNU LGPL version 3 to encourage uptake and reuse.

### Library development

The SpeckTackle library consists of several files to structure the project and simplify development. The library can be built using a Make script. Modules required for building and ‘minifying’ the project are listed in the online documentation. The SpeckTackle CSS (st.css) is required in addition to the library (st.min.js) to control the style and layout of charts.

SpeckTackle provides pre-defined chart types for IR, MS, NMR (1D and 2D) and general time series data. The layout and mouse behaviour of each chart type is defined by *de facto* standards and concern the x- and y-axes (placement/direction), zoom behaviour (box/range-zoom), color schemes, and representation of data points (impulse/line/point). One example for a typical mouse behaviour is resetting the zoom by double-clicking on the chart.

A custom chart type extends the base chart, which defines a two dimensional Cartesian coordinate system and box-zooming as default mouse behaviour. At a minimum, a custom chart needs to implement three functions that describe how data is to be drawn (1) and how the x- and y-values (2,3) are scaled, e.g. linear or logarithmic. Further customisation to the base chart are achieved by extending or overriding existing base chart functions.

Expected options such as a chart title, x- and y-labels, an interactive legend, chart margins, and signal labels can be set on chart creation in a cascading fashion before a data set is assigned to the chart.

SpeckTackle accepts input in JSON format either directly or through Ajax. Similar to the pre-defined chart types, data handlers are implemented to reduce library set up to a minimum. A data handler is used to describe the structure of input data and to deal with data load and removal events after the data handler is associated (bound) to the chart. It should be pointed out that the library is stateless, i.e. files are reloaded and the library is reset when the user navigates away from the website.

**Figure 1:**
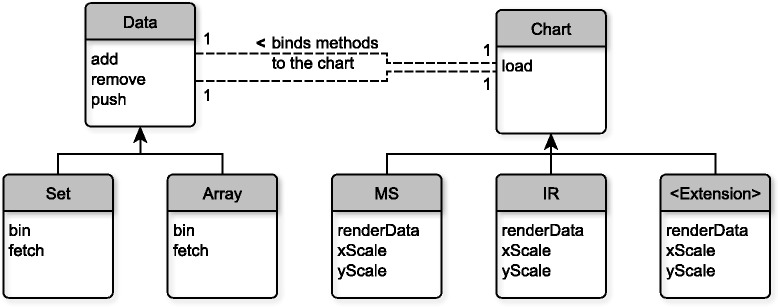
Simplified diagram of the relationships between the data and chart structures and their extensions. This diagram illustrates the concept of how the data to be displayed and the chart type that displays the data are separated and how they can interact. Predefined or new custom data and chart structures extend their individual *Data* and *Chart* objects, i.e. they inherit a contract that defines how the data and chart structures can be accessed (black line). Data extensions need to provide a *fetch* and *bin* function that define how the data is retrieved from a resource and how it is binned whereas chart extensions need to define how the data is rendered (*renderData*) and how the x- and y-values are scaled (*xScale*, *yScale*). Depending on the function definitions, chart extensions can for example appear as MS charts or IR charts. In general, a chart *extension* can be written to represent many other types of two dimensional charts if the contract is fulfilled. When the data object is loaded to the chart object (dashed line), its *add* and *remove* methods are associated with the chart. Each chart can only have one data handle associated with it as indicated by the number ‘1’. The data object serves as data handle for all data-related interactions.

As a general rule, all interaction between a chart and raw data is mediated through a data handler, which keeps track of added data series and their properties. Multiple data series (overlays) are supported with the ability to highlight an individual data series via its legend key. Figure 1 illustrates the above described concepts and relationships. A detailed description of the individual functions is provided in the online documentation.

The JavaScript code listing below provides a minimalistic, commented example of steps required to set up a chart for mass spectra and load data. Cysteine [MTBLC15356] from the MetaboLights website is used as example.



A data handler also controls how data is binned: for larger data series, e.g. NMR spectra with >60,000 points, the visualisation of all data points is unnecessary and slows down response times of a chart. Instead, a data handler can bin data series by their minimum, e.g. for IR spectra, or maximum signal intensities, e.g. for NMR spectra, for a given bin width – typically one pixel – and x-axis scale. Binning is carried out on data load and can be adjusted – or turned off – in the library. The default of one pixel, which typically provides enough resolution to show the shape of a data set, is controlled by the variable*binwidth* in the *bin* method in its respective data object. The chart type defines whether data is binned by minimum or maximum.

Annotations and tooltips for data point selection events are supported through the concept of annotation types. Implemented annotation types encompass textual annotations that are drawn onto the chart and textual/structural tooltip annotations. Whereas textual annotations are simply drawn besides their target data points, tooltip annotations are specified as key-value pairs

In the first case, the key-value pairs are character strings that are displayed as list in the form ‘ <*key*>: <*value*>’.

In the second case, the value of each pair is treated as URL to a MDL Molfile, which contains the molecular structure to be displayed, and resolved accordingly. SpeckTackle provides its own internal MDL Molfile parser and draws molecules as SVG directly from the file.

The following two code listings demonstrate the concept. Annotation type names and the function name *annotationColumn* have been abbreviated in the interested of space. The structure of the annotation JSON file is defined in the data handler before data can be loaded.



The annotation JSON file contains two required columns by default: the first column defines the group. Multiple groups are permitted and are listed for selection on data load. The second column defines the lookup value in the x-domain. Subsequent columns must match the structure described to the data handler.



## Results and discussion

JavaScript is one of the most popular and widely adopted programming languages on the web that has been successfully applied to the problem of data visualisation in many projects. JavaScript is therefore the technology of choice for a cross-browser charting library and has been chosen for this project. Additionally, the authors have chosen a modular project structure with powerful and reliable dependencies in the background to ensure that SpeckTackle can easily be maintained and extended.

D3 and JQuery are selected as dependencies for SpeckTackle because they are proven tools for DOM traversal and data visualisation. They ensure simplicity and robustness of the library by enabling developers to focus on the core problem of visualising a particular type of data. Whereas D3 and JQuery support other elements besides SVG, such as canvas, the library is build around HTML5 and SVG. SVG performs well for the majority of spectra use cases and can easily be styled by the end user via CSS. In addition, the SVG DOM facilitates the development of interactive charts by the ability to bind browser events to SVG elements and manipulate SVG elements using JavaScript. As a consequence, only browsers that support HTML5 and SVG standards are compatible with SpeckTackle, which represent the majority of web browsers in use.

Default chart types and data handlers are provided to reduce library set up to an absolute minimum. The ‘look and feel’ of MS or NMR spectra are well established and are reflected in the default chart types for these technologies, e.g. box zooming for MS charts and range zooming for NMR charts.

**Figure 2:**
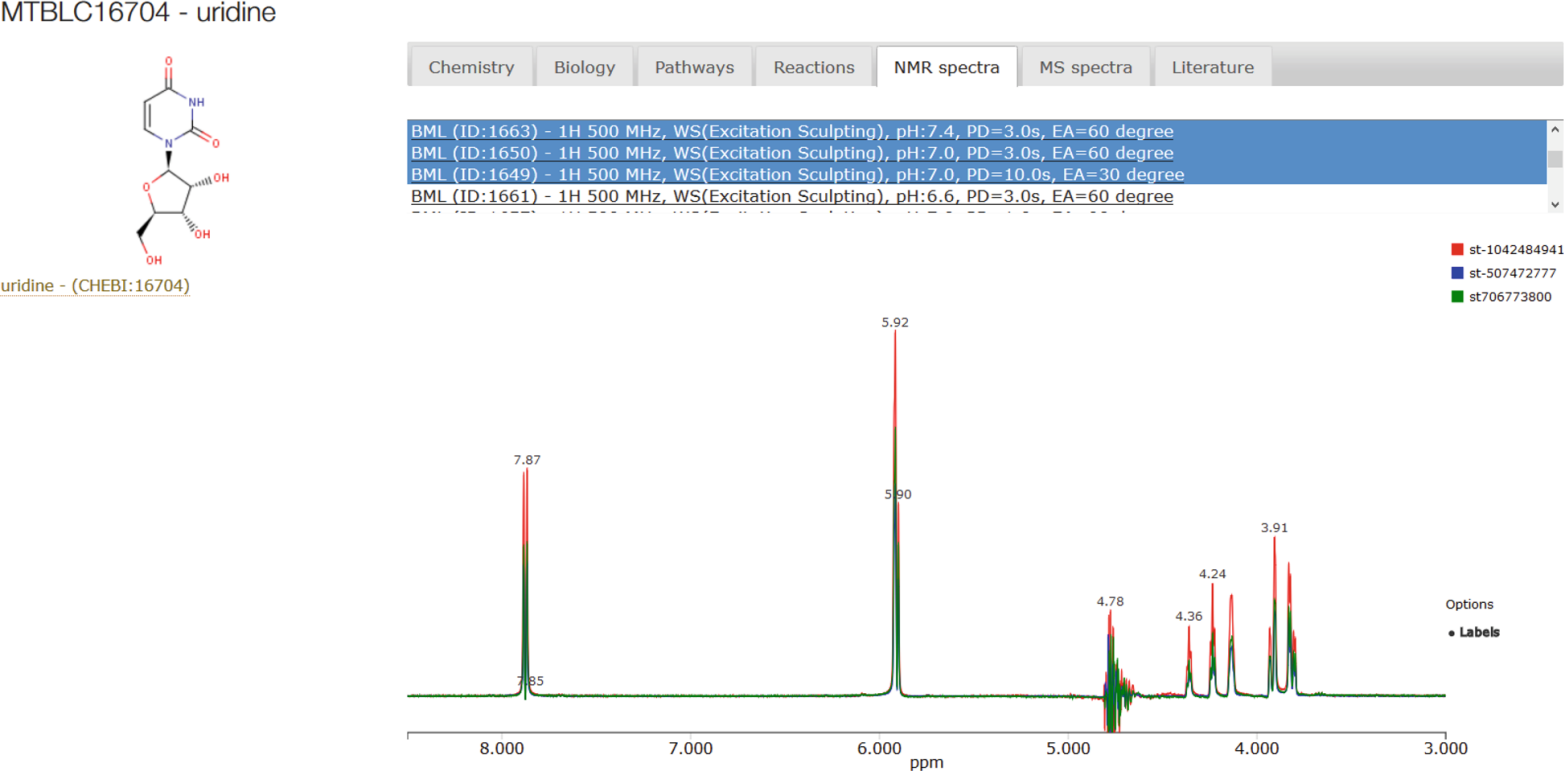
Screenshot of the SpeckTackle viewer integrated into the MetaboLights database. An overlay of three 500 MHz NMR spectra (acquired with different parameters) of Uridine [MTBLC16704] is shown between 3 ppm and 9 ppm. Chart series names for the optional chart legend in the upper right hand corner were replaced by computed identifiers because they were not provided on data load. The option ‘Labels’ was turned on for signal labels.

The use of default chart types custom-tailored to the life sciences and its portable design makes SpeckTackle particularly appealing to the bio- and cheminformatics communities that require a solution to their data visualisation needs, e.g. in browser-based front-ends of database. The ability to browse a spectral reference library, quickly visualise a spectrum or display difference charts, e.g. of spectra queries run on a website, can be immensely helpful in a decision making process. As a test case, the charting library has been integrated into the MetaboLights website, which was greatly facilitated by the pre-defined chart types and data structures. Figure 2 shows a screenshot of the viewer with three superimposed NMR spectra of the metabolite Uridine [MTBLC16704] as integrated in the MetaboLights website. The viewer enables quick comparisons of available reference spectra for that compound, e.g. to gauge the quality of the reference spectra. In other cases, such as for tandem MS spectra, reference collections could be screened to inspect the number of fragment signals.

**Figure 3:**
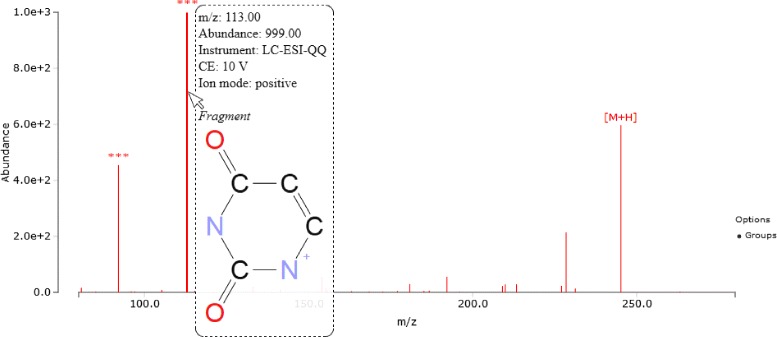
Screenshot of the SpeckTackle viewer with annotations and tooltips. A fragmentation spectrum (MS^2^) of Uridine [MTBLC16704] is shown. Annotations are displayed for the main peak and two fragments. Tooltips for individual signals are loaded on mouse over. The putative fragment structure plus additional textual information is shown for the signal at *m/z* =113.00.

Basic functions such as labels or highlights on mouse over events are covered by the library and are easy to understand and modify if required. To ensure wide uptake within the community, a flexible annotation framework is implemented that can show custom tooltips and annotations. For example, Figure 3 shows a MS spectrum of Uridine with tooltip information for the uracil fragment. The data and structure of the tooltip are described in the preceding section. The ability to display additional information such as the exact *m/z* value, the fragment associated with a signal or references from other sources of information provide the context required to greatly facilitate a user’s understanding of the data.

## Conclusions

We have described the design, implementation, and functionality of the JavaScript charting library SpeckTackle. The library is released under GNU LGPL to encourage uptake and reuse within the community. SpeckTackle provides several pre-defined chart types with fine-tuned ’look and feel’ for different kinds of spectroscopy instruments. It is easy to integrate into web-facing resources and covers common visualisation tasks to make it fit-for-purpose across online resources.

The SpeckTackle library is small (∼46 KB) and additional default chart types can easily be added. The latest version of the library including examples and documentation is available online in its public repository. We hope that the charting library finds wide-spread adoption within the community and simplifies the development of web-facing resources.

## Availability and requirements

- **Project name:** SpeckTackle
- **Project home page:**https://bitbucket.org/sbeisken/specktackle
- **Operating system(s):** Platform independent
- **Tested web browsers:** Firefox (v34), Internet Explorer (IE9+), Opera (v26), Chrome (v39), Safari (v7.1)
- **Programming language:** JavaScript
- **License:** GNU Lesser GPL version 3
- **Any restrictions to use by non-academics:** none

## Additional file

Additional file 1
**SpeckTackle_Demo.html.** HTML web page demonstrating different chart types and functions of the SpeckTackle library. Example data is embedded in the web page.
