# Supplementary material for: SpeckTackle: JavaScript charts for spectroscopy
Source: J Cheminform. 2015 May 9;7:17. doi: 10.1186/s13321-015-0065-7 (PMC4432097; doi:10.1186/s13321-015-0065-7)
Supplement: Additional file 1 — SpeckTackle_Demo.html. HTML web page demonstrating different chart types and functions of the SpeckTackle library. Example data is embedded in the web page. [file 13321_2015_65_MOESM1_ESM.zip › index_complete.html]

SpeckTackle


- MS I
- MS II
- NMR
- NMR2D
- |
- Chromatogram
- Difference Chart
- Spectral Match
- →
- Add Data

---
